# Some Functional Disorders of the Heart

**Published:** 1891-02-21

**Authors:** 


### The Practitioner's Mirror and Retrospect.

[*The Editor will be glad to receive offers of co-operation and contributions from members of the profession. There is no doubt that much valuable material full of interest and instruction is at present lost to science. This is largely so in the case of* (1) *the younger and less-known members of the hospital staff* (2) *those connected with cottage hospitals, and* (3) *the great body of medical practitioners not attached to any institution. The co-operation of all is cordially invited to enable the Editor to make* The Hospital *of the utmost possible use and value to the profession. Specialists willing to review books on their special subjects are invited to communicate this fact at once. All letters should be addressed to* The Editor, The Lodge, Porchester Square, London, W.]

### MEDICINE.

### SOME FUNCTIONAL DISORDERS OF THE HEART.

Many of the most valuable discoveries in the science of medicine have been made by examination of what have been termed residual phenomena. The results of known causes must be carefully estimated, after which the consideration of additional or excessive symptoms leads often to our finding hitherto unsuspected and important factors. In organic diseases of the heart the alteration in rate and rhythm are frequently more than the morbid process will well account for, and there are a whole series of cases where the alterations are at least as great, while the lesions become less and less, till at last none whatever can be detected. We are led then to suspect a fruitful field of research in connection with those numerous instances of tachy and brachy-cardia and irregular pulse which we are apt to dismiss as merely functional. Dr. Bristowe has well described many cases of "recurrent palpitation of extreme rapidity, in persons otherwise apparently healthy," and he includes in his collection both instances of simple rapid action and those where irregularity is added. Such cases occur by no means rarely. The beats will often reach 200 or even 300 per minute, the rapidity commencing suddenly, and lasting for hours or days, when a sudden fall may occur, accompanied by great feelings of relief. Or the pulse may maintain a persistently high rate. Some such cases have gone on for five, ten, or eighteen years, but are apt to be followed by dilatation, lung troubles, or, as in a case we have recently watched, by thrombosis and gangrene of a limb. In some of Dr. Bristowe's cases there was a history of rheumatism or syphilis, and Buveret and others have noticed their occurrence after overstrain, but in a large number of instances no cause whatever could be discovered. Dr. West would refer these symptoms to myocarditis, but it is clear that at times, as Buveret shows, no trace of any lesion is apparent on the post-mortem table either in the muscle, valves, or nerves. In the interesting discussion at Birmingham, Sir Dyce Duckworth insisted that many of these cases were connected with an incipient organic disease dependent on the same nerve lesion which he believes to be present in chronic rheumatic arthritis. Dr. Kent Spender has drawn attention to the existence of a persistently rapid pulse in the earlier stages of this so-called rheumatic affection, but the paroxysmal form of tachycardia has certainly not been observed here. Women too, are affected more than men, which is not the case in the latter affection.

Temporary acceleration of the heart from exercise or emotional causes is, of course, common enough both in the healthy and in the diseased. Easily produced, it quickly passes away in the healthy heart. Dr. Pye Smith, in his Hunterian address, points out that "we have no corresponding means of slowing a man's pulse below its natural frequency, when he is lying down tranquil in mind and body." An elaborate account of a case where the power of voluntary retardation of the pulse seems to have occurred was long ago chronicled by Cheyne, the author of "The English Malady," and another has quite recently been examined and described, while Czermak's well-known experiment, by compressing his own vagus, is said to have been repeated with success as a therapeutic agent in a few cases of paroxysmal heart hurry under Buveret. Practically, however, Dr. Pye Smith's remark remains true, and should not be passed over.

In organic disease of the heart, on the other hand, a persistently rapid pulse is met with frequently, and may be wholly or in part ascribed to the lesion. We have first, the cases where an altered state of the blood interferes with the heart's action, as probably in phthisis, pyrexia, or anæmia; or secondly, where a mechanical hindrance to the propulsion of the blood occurs, whether it be a valvular lesion, a displacement from effusion or a distended stomach, or a blocking of the peripheral circulation as in bronchitis and in certain stages of granular kidney. In a third class we find only nerve influence at work. This may be a reflex action on the vagus from dyspepsia or uterine derangements, or a direct result of tubercular deposit in later periods of meningitis. The palpitation in exophthalmos has been ascribed either to an affection of the vagus or sympathetic ganglia, and it appears at times to constitute the sole symptom. In Tabes, again, cardiac crises have been occasionally observed, and here we are at least justified by analogy in supposing a nerve lesion. We have said that the same has been asserted for osteo-arthritis, and, indeed, a nerve agency has been claimed for the numerous cases of rapid heart persisting after mild attacks of influenza. Yet to refer symptoms to an affection of the nervous system is often little else than a confession of our absolute ignorance of their cause. No doubt, where in healthy hearts an emotional influence produces a temporary acceleration, we are justified in referring it to an inhibition of some part of the regulating nervous mechanism, just as we do in the opposite case of a frog whose heart is brought to a standstill by a blow on the abdomen. But in the cases of persistent or paroxysmal heart hurry where none or an insufficient organic lesion can be discovered by the most minute search, we must carefully consider the possibility of some muscular or mechanical difficulty in the circulation as well as that of an unprovable nerve affection. Our meaning is well illustrated by the growth of opinion on the subject of "irritable heart," first described by Da Costa from observations made in the American war, and afterwards by Thum, Leyden, and Fräntzel. Quite recently Sajkovic has discussed similar instances occurring in the Servo-Bulgarian war, and English military surgeons have recognised the affection as not uncommon among ourselves. It occurs chiefly in young soldiers subjected to unwonted privations, heavy drill, and severe exertions and strains. There is a rapid irregular pulse and violent action of the heart without any valvular lesion. It is significant that it is seen more rarely in sailors, whose dress causes less mechanical embarrassment to the circulation, and whose training is more gradual, and who also are much less subject to aortic aneurysm. Rest brings about a cure more often than any other remedy, though digitalis and tonics appear to have some value. Here overstrain seems to be the cause of the disease. It very quickly produces hypertrophy, which may disappear as rapidly as it came, often leaving no sign to account for the irregular action, which may continue for a longer period. In other instances hypertrophy may be followed or replaced by dilatation. These varying changes in the cardiac muscle are so rapidly and commonly induced by overstrain that we must probably regard them, and not any emotional or central affection, as lying at the root of the mischief. If this is so, we have an explanation in the muscular mechanism of the heart for what seemed a purely nervous disorder. It is noteworthy in passing how little acceleration of the pulse is produced in parturient women during the course of normal labour.

In an organ whose powers of repair are so rapid and great, we cannot be surprised at the frequent absence of anatomical lesions. Early and careful examination is therefore of the utmost importance for throwing light on the production of these functional disorders, which, once understood, would probably explain much that is now obscure in the working of organic diseases. Some help might be gleaned by careful comparison with the results of disordered action of other involuntary muscles, where the mechanism is less complex. We do at least see that a persistently accelerated peristaltic action may be set up as a habit by external causes in otherwise healthy organs, and remain long after the cause has been withdrawn. It would be interesting, too, to consider the reasons for the greater frequency of functional disorders in the heart over those in the lungs, bladder, and digestive tract. The investigations of Gaskell have paved the way to the explanation of much that is obscure and complex in cardiac innervation. "Under certain circumstances, stimulation of the vagus may produce, not inhibition, but either augmentation of the beats or quickening of the rhythm, or both, and Gaskell sees reason to think that the same fibre may cause these different effects according to the condition of the muscle at the time, and that, on the whole, the vagus is a constructive and not a destructive nerve, producing greater functional activity, though this is sometimes preceded by a diminution of function." Roy, too, has shown that the heart in systole always contains some residual blood, and that this rapidly increases under overstrain.

The therapeutic interest of functional disorders is very great, though hitherto remedies having a known influence on either nerve tissues or on the heart muscles have been found of little, if of any, use. In the Birmingham discussion this was well brought out. Sir Walter Foster mentioned that large doses of quinine continued for a long period had alone been found to possess any therapeutic value in many cases of idiopathic tachycardia. The majority, probably, of such cases as those described by Dr. Bristowe resist all attempts at medication, and yet there is certainly no marked sclerosis or degeneration such as would account for the powerlessness of ordinary drugs As much has been done in writer's cramp by regulated drill, combined with rest, so Oertel's scheme of cardiac gymnastics has been found of use in certain cases of irregularly acting hearts. The icebag laid on the præcordia, or the induced current applied over the vagus, may now and then give some relief, and even Czermak's method of compression, as Buveret claims, may be occasionally applicable; but till we have more certain knowledge on the causation of these states, both as to what factors can be excluded and as to those which are, or have recently been present, we must remain in doubt as to the possibilities of curative treatment. We repeat that in the study of functional disorders of the heart there is a wide field for discoveries which should be fruitful, not only in their application to these disturbances, but also in the elucidation of organic diseases where similar symptoms are indirectly produced.

